# Correlation between increased flushing intervals and malfunction and infectious complications in fully implantable catheters during the COVID-19 pandemic

**DOI:** 10.31744/einstein_journal/2024AO0736

**Published:** 2024-11-21

**Authors:** Alexandre de Oliveira Esteves, Vitor Lauar Pimenta de Figueiredo, Glauco Fernandes Saes, Antônio Eduardo Zerati, Pedro Puech-Leão, Nelson Wolosker, Nelson De Luccia

**Affiliations:** 1 Universidade de São Paulo Faculdade de Medicina São Paulo SP Brazil Faculdade de Medicina, Universidade de São Paulo, São Paulo, SP, Brazil.; 2 Hospital Israelita Albert Einstein Faculdade Israelita de Ciências da Saúde Albert Einstein São Paulo SP Brazil Faculdade Israelita de Ciências da Saúde Albert Einstein, Hospital Israelita Albert Einstein, São Paulo, SP, Brazil.; 3 Hospital Israelita Albert Einstein São Paulo SP Brazil Hospital Israelita Albert Einstein, São Paulo, SP, Brazil.

**Keywords:** Catheters related infections, Catheters, indwelling, Vascular access devices, Flushing, Long term care, Saline solution, COVID-19, Coronavirus infections, Pandemics

## Abstract

**Objective:**

To evaluate the incidence of malfunction and colonization rates of fully implantable long-term catheters left unflushed during the COVID-19 pandemic; and to evaluate the average cost of transporting each patient to the hospital for flushing.

**Methods:**

During the COVID-19 pandemic, patients reduced the number of hospital visits and stopped flushing their catheters periodically with saline solution. After the pandemic stabilized in 2022, patients who completed chemotherapy treatment had their long-term catheters removed. We evaluated the catheters’ function and colonization rates. To evaluate the incidence of malfunctions and the colonization rate of these catheters, we tested the flow and reflux during removal surgery, before removal, and by culturing their tips. These catheters were divided into two groups: a standard group, in which the last flushing occurred before 90 days, and another group, in which the last flushing occurred after 90 days. We analyzed the correlation between the time at which these catheters were closed, the incidence of malfunction, and the colonization rate of these catheters. To avoid confusion due to the reduced sample size, a second analysis was performed between the group of catheters that did not work and those that worked, evaluating the time they were closed and the catheter tip culture. We also analyzed the financial costs for each patient from home to the hospital.

**Results:**

Among the 66 patients included in the study, 28 spent >90 days without catheter flushing, and 38 spent <90 days. The incidence of infection occurred in two patients with >90 days of flushing and in three patients with <90 days of flushing. Catheter malfunction occurred in 4 patients in the group with >90 days without flushing and in 5 patients with <90 days of flushing. In the secondary analysis, the group with a functioning catheter (n=52) had a mean time of 152 days, whereas for the group with a non-functioning catheter (n=9), the mean time was 229 days (p=0.51). No differences were statistically significant. No correlation was found between the colonization rate of catheter in the group with a functioning or non-functioning catheter, as the group with a functioning catheter had three cases of positive catheter tip culture and the group with a non-functioning catheter had one case of positive catheter tip culture. The average cost for each patient to travel from home to the hospital was 39.01 reais (approximately 7.50 USD).

**Conclusion:**

Among the patients followed up at our hospital during the COVID-19 pandemic, no statistically significant difference was observed in the function and colonization rate of long-term catheters between those who underwent flushing at intervals of <90 days and those with intervals of >90 days.

## In Brief

We retrospectively analyzed the effect of increased intervals between port flushings in oncologic patients who maintained their port-o-caths without flushing during the COVID-19 pandemic. Patients were divided into two groups: those with >90 days without flushing and those with <90 days. Aside from complications, we observed a socioeconomic impact.

## Highlights

■ Long-term catheter flushing for >90 days has preserved function.
■ Ports cleaned after 90 days have the same colonization rate as those flushed early.
■ Long-term catheters may be colonized in asymptomatic patients.
■ The longer port cleaning interval reduces costs.

## Graphical abstract

**Figure:**
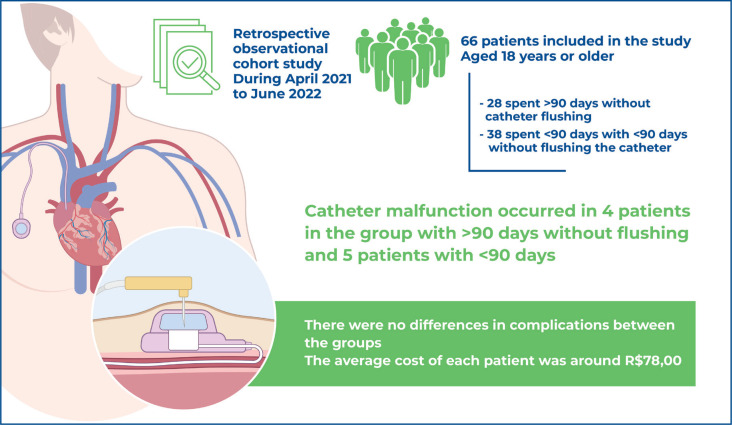

## INTRODUCTION

A fully implantable long-term catheter was originally described in 1963 by Ommaya^(1)^ as a cerebrospinal fluid reservoir and manual pump.

This device was first used to facilitate repeated injections of drugs into the cerebrospinal fluid in patients with fungal meningitis.^(1)^ Over the years, it has been developed and used in the treatment of malignant neoplasms of the nervous system, allowing the perfusion and instillation of cytotoxic agents.^(2)^

Since then, these catheters have been of great importance in oncological scenarios as they provide access to the deep venous system in a practical, easy, and long-lasting manner. The infusion of vesicant chemotherapy drugs is intermittent and continuous, with low complication rates.^(3,4)^

Despite being a safe device, the simple fact that the catheter remains in the lumen of the vessel for long periods can lead to complications. The most frequently described are infections, venous thrombosis, catheter occlusion, poor tip positioning, migration, and malfunction.^(5)^

To ensure patient safety and limit the number of potential catheter-related complications, catheter manufacturer guidelines recommend that fully implantable long-term catheters undergo monthly flushing^(6–8)^ until removal of the catheter, which can take years depending on the period of surveillance required for the oncological disease.

However, the emergence of the coronavirus disease (COVID-19) pandemic in December 2019 in China meant that we avoided exposing patients to hospital environments as much as possible.

For this reason, the Cancer Institute of the State of São Paulo (ICESP - *Instituto do Câncer do Estado de São Paulo*), as part of the *Hospital das Clínicas* of *Faculdade de Medicina* of *Universidade de São Paulo* (HCFMUSP), had to interrupt the flushing of the fully implantable long-term catheters since the HCFMUSP had become one of the most important COVID care centers in São Paulo. In addition, the increased risk of developing severe COVID when patients with cancer are infected with SARS-CoV-2 has led to the restriction of face-to-face consultations in outpatient clinics.

With the stabilization of the pandemic and the emergence of vaccines against SARS-CoV-2, patients began to return for face-to-face consultations at the HCFMUSP and ICESP. Consequently, cases in which the neoplasm was treated and patients had completed the oncological disease surveillance period with no evidence of active oncological disease were referred for the removal of fully implantable long-term catheters.

At that moment, we recognized the opportunity to address two unanswered questions in the current literature: whether long-term fully implantable catheters, which are left for long periods without maintenance flushing, stop working, and if they have the same rate of bacterial colonization in asymptomatic patients as those with routine maintenance flushing.

To answer these questions, the catheters were tested for their functioning during the catheter removal procedure, and after removal, the catheter tip was sent for culture.

In an attempt to extract the maximum amount of information that the pandemic situation imposed on us about the stoppage of catheter maintenance flushing, we also raised the financial costs that these patients had with transportation to the ICESP to undergo their routine flushing of the catheters, which evaluated the impact of periodic catheter flushing for both the patient and the institution.

## OBJECTIVE

To evaluate the incidence of malfunction and the colonization rates of fully implantable long-term catheters left unflushed during the COVID-19 pandemic. Evaluate the average cost of transporting each patient to the hospital for flushing.

## METHODS

### Study design

We conducted a retrospective observational cohort study to assess the correlation between the number of days from the last saline solution flush until catheter removal and the prevalence of catheter colonization, as measured by catheter culture and functionality before catheter withdrawal. Consent was obtained from all the participants at the time of catheter removal. This study was a branch of a larger project at the institution that was approved by the ethical review board of *Faculdade de Medicina, Universidade de São Paulo* (CAAE: 40009114.1.0000.0065; #974.501).

To assess the social and financial impacts of periodic flushing of catheters, a questionnaire was used to evaluate patients’ travel time to the ICESP, the means of transport used, and the financial cost of transportation.

### Patients and participating center

Patients aged 18 years or older who used a fully implantable long-term catheter, who were being followed up at the ICESP, and who removed the catheter between April 2021 and June 2022, with proper culture of the catheter tip, were eligible for the study.

In our hospital, the catheter undergoes flushing with saline solution every 90 days after the completion of chemotherapy until it is removed. The technique involved puncturing with a Huber needle after adequate asepsis using chlorhexidine. This procedure was performed by an oncology-specialized nurse wearing appropriate protective gear, including a cap, mask, and sterile gloves, in addition to the placement of a fenestrated field.

Following puncture, 5mL of fluid was aspirated and discarded. Subsequently, 20mL of saline solution was infused, and the puncture site was sealed at the end of the infusion to avoid blood reflux into the catheter.

During the COVID-19 pandemic, this routine was interrupted, and many patients remained for periods without catheter flushing, which allowed us to conduct the study and analyze these catheters.

During surgery, to remove the catheter after asepsis, antisepsis, and placement of sterile drapes, the catheter was punctured with a Huber needle, and the presence of flow and reflux was assessed. The attending and resident physicians performed this assessment.

### Statistical analysis

To analyze the impact of not flushing these catheters periodically, we divided the patients into two groups: those who had the last catheter flushing in a period of <90 days, and those who had their catheter flushing in a period greater than 90 days until its withdrawal. To avoid confusion due to the reduced sample size, a second analysis was performed between the group of catheters that did not work and those that worked, evaluating the time they were closed and the catheter tip culture. We also analyzed the financial costs for each patient, from home to the ICESP.

To analyze the frequency of events (types of cancer, presence of infection, and sex), the Ç^2^ test was used, and the absolute values and percentages were acquired. For continuous variables such as age and length of days, the catheter was closed, and a *t*-test was used. For all analyses, a p<0.05 was accepted as statistically significant. Data were analyzed using Jamovi software (v.1.0.7, The Jamovi Project).

## RESULTS

### Patient and demographic data

This study included 66 patients who underwent catheter removal and culture between April 2021 and June 2022. Five patients were excluded from the malfunction assessment (3 in the >90-day group and 2 in the <90-day group), and three patients were excluded from the catheter tip colonization analysis because of a loss of data.

The average age of the patients was 48.3 years, and the majority were female (72.7%). The most common cancer was breast cancer (30.3%), followed by Hodgkin lymphoma (22.7%). The average interval between catheter flushing and removal was 159.7 days.

### Incidence of catheter malfunction

Table 1 presents data from patients who had their catheters closed for >90 days without flushing. Of these patients, 21 had normal catheter function, and only 4 did not. Most of these patients were women, with an average age of approximately 50 years. Breast cancer and Hodgkin's disease are the most prevalent types of cancer. The mean duration for which the catheter remained closed ranged from 299 to 593 days. Notably, no difference was observed in any variables between the groups that maintained catheter function and those that did not (p>0.05).

**Table 1:** Patients with >90 days without flushing the catheter

		Functioning (n=21)	Not-functioning (n=4)	p value
Gender (women), n (%)		16 (76.6)	4 (100.0)	0.27
Age (years)		49.4 ± 16.6	46.3 ± 2.75	0.71
Closed time (days)		312 ± 229	450 ± 593	0.41
Diseases, n (%)				
	Breast câncer	6 (28.6)	2 (50)	0.48
	Hodgkin's disease	5 (23.8)	0 (0.0)	
	Other	10 (47.7)	2 (50.0)	

Data presented as mean ± standard deviation as frequency (absolute and relative).

Table 2 presents the same variables for patients who underwent <90 days after surgery without catheter flushing. The results were similar to those shown in table 1, except for the time at which the catheter was closed (p>0.05). As expected, a shorter duration was observed in this study, ranging from 25 to 51 days.

**Table 2:** Patients with <90 days without flushing the catheter

		Functioning (n=31)	Not-functioning (n=5)
Gender (women), n (%)		21 (67.7)	5 (100.0)
Age (years)		44.8 ± 17.5	49.6 ± 12.6
Closed time (days)		43 ± 25	51 ± 26
Diseases, n (%)			
	Breast cancer	5 (16.1)	1 (25)
	Hodgkin's disease	14 (45.2)	1 (25.0)
	Other	12 (38.7)	2 (50.0)

Data presented as mean ± standard deviation as frequency (absolute and relative).

Table 3 presents the values of patients with functioning and non-functioning catheters, regardless of the flushing time (n=61). Similar patterns were also observed here, except for the time at which the catheter was closed. For the group with functioning catheters (n=52), the mean time was 152 days, whereas for the non-functioning group (n=9), the mean time was 229 days (p=0.51). Although a difference between the two groups was observed, it was not statistically significant (p>0.05).

**Table 3:** Total amount of patients with functioning and not-functioning catheters

		Functioning (n=52)	Not-functioning (n=9)	p value
Gender (women), n (%)		37 (71.2)	9 (100.0)	0.13
Age (years)		46.7 ± 17.2	48.1 ± 9.2	0.56
Closed time (days)		152 ± 197	229 ± 420	0.51
Diseases, n (%)				
	Breast cancer	11 (21.2)	3 (37.5)	0.38
	Hodgkin's disease	19 (36.5)	1 (12.5)	
	Other	22 (42.3)	4 (50.0)	

Data presented as mean ± standard deviation as frequency (absolute and relative).

### Colonization rate of the catheters

**Figure 1:**
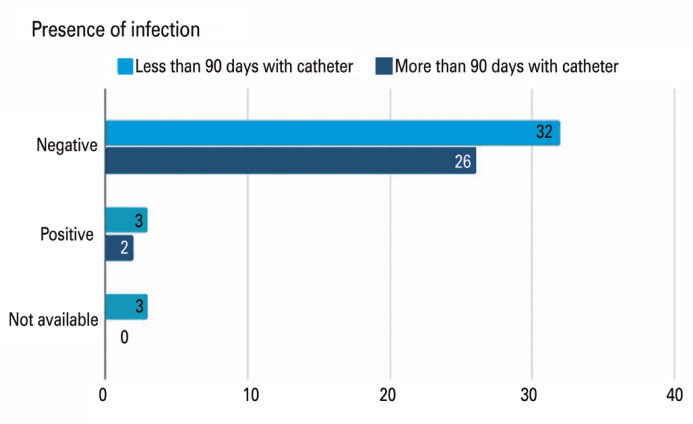
Presence of positive catheter tip culture in patients with >90 days and <90 days with catheter

**Figure 2:**
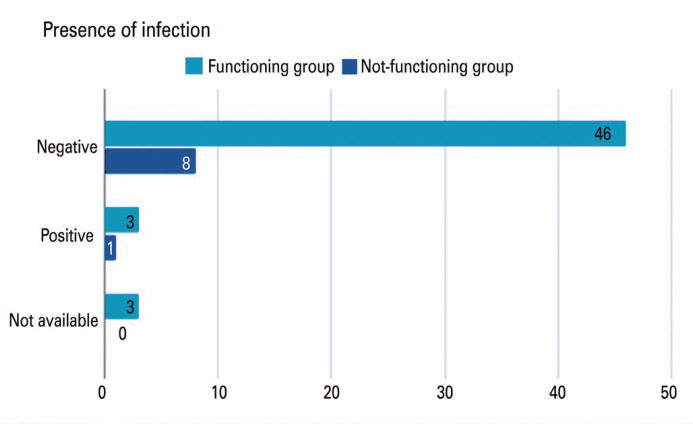
Presence of positive catheter tip culture in patients with functioning and not-functioning catheters

Figures 1 and 2 show the catheter colonization rates for these groups. Regardless of the number of days the catheter was closed (Figure 1), the number of colonizations by these catheters was low. Three cases of positive catheter tip culture occurred in the group with <90 days, and two in the group with >90 days of culture (p>0.05). Figure 2 shows that the colonization rates of the functioning and non-functioning groups were similar. No difference was observed between the groups; the functioning catheter group presented three cases of positive catheter tip culture, while the non-functioning catheter group presented one case (p>0.05).

### Assessment of socioeconomic costs

Table 4 displays the transportation cost and average time spent by patients to reach the ICESP, as well as the frequency with which they went to the ICESP with the catheter and after its removal. The average cost of a one-way trip for each patient was R$39.01, and the average time spent was 101.5 minutes.

**Table 4:** Financial costs and time spent to reach ICESP (health service responsible for flushing the catheters)

	Cost of transport one-way (real)	Time spent to reach ICESP	Annual frequency to ICESP without catheter	Annual frequency to ICESP with catheter
Valid	28	30	6	29
Missing	38	36	60	37
Average	39.0	101.5	2.5	15.3
Median	15.5	90	2.0	12.0
Standard deviation	64.6	84.1	1.4	26.4

ICESP: *Instituto do Câncer do Estado de São Paulo*.

The frequency of visits to the ICESP is 2.5 times per year after catheter removal and 15.3 times per year with the catheter in place.

Table 5 displays the main means of transportation used by patients to travel to the ICESP, with the most common being by car (36.7%), followed by subways and buses (16.7%). Most patients (60%) used public transportation to arrive at healthcare institutions.

**Table 5:** Means of transport used by patients to get to ICESP

Transportation	Frequency	Percentage %	Valid percentagem %
Walk	1	1.5	3.3
Car	11	16.7	36.7
Car and bus	1	1.5	3.3
Subway	2	3.0	6.7
Subway and train	1	1.5	3.3
Subway and bus	5	7.6	16.7
Subway and bus and train	1	1.5	3.3
Subway or bus or App car	1	1.5	3.3
Subway, monorail and App car	1	1.5	3.3
Van	1	1.5	3.3
Bus	3	4.5	10.0
Bus or train	1	1.5	3.3
Bus or App car	1	1.5	3.3
Missing	36	54.5	
Total	66	100.0	

ICESP: *Instituto do Câncer do Estado de São Paulo*.

## DISCUSSION

Long-term catheter use is essential for infusion therapy in cancer patients.^(9)^ However, its use still presents some obstacles, such as possible late complications^(5)^ and the need for monthly maintenance flushing of the catheter,^(9)^ according to the manufacturers’ guidelines. Despite these recommendations, the ideal period for maintenance flushing is not yet fully understood.^(10)^ Periodic monthly flushing, guided by the manufacturer's manual, gave way to quarterly flushing in many oncology services.^(11)^

The need to ensure that the port is kept flushing frequently is financially burdensome, both for the hospital and the patient, with travel and food costs. In addition, the time spent on this process affects the patient's quality of life.

However, the benefits generated by periodic port maintenance flushing do not seem to be significant.^(11)^ Our retrospective analysis of 66 patients who used the catheter and were followed up at the HCFMUSP found no significant difference (p>0.05) in the colonization rate between those who underwent flushing at intervals of <90 days and those with intervals of >90 days. This is similar to the results of Ignatov et al., who did not show an increase in the infection rate in the group with a flushing interval of >12 weeks, with the exception that we had a longer period than theirs. Similar results have been reported by Fornaro et al., Kefeli et al., and Palese et al.^(12–15)^

Of these patients, only one patient in the group that underwent flushing in <90 days had thrombosis after 34 days of treatment, and the catheter did not work. This result is in line with other studies,^(10,11,16)^ which suggest that extending the maintenance flushing period does not interfere with the efficacy or complication rate of catheters.^(10,16)^

Another important point is that, unlike the other studies, these patients were subjected to a different therapy in the maintenance flushing of the catheters, making only the infusion of 20mL of saline solution and without the concomitant use of anticoagulants or fibrinolytics in the infused solution, as in other published papers, such as those by Ignatov et al. and Fonaro et al., who placed heparin solution inside the catheters.^(12,13,16)^

Another relevant fact was that all tips of the removed catheters were sent for culture, and the growth of >15 colonies on the plate was considered a positive semiquantitative culture.^(17)^ None of these patients had catheter-related blood infections; however, even clinically asymptomatic patients (without chills, fever, or malaise during catheter manipulation) had their tips removed and sent for culture. Some studies in medical literature did not routinely send catheter tips for culture. Using these methods, we observed that in the group of patients with catheters with a maintenance flushing interval of <90 days, three catheters had a positive culture for *Staphylococcus aureus*, which is the etiological agent most often noticed in cases of catheter infection.^(18,19)^ Their catheter remained closed for an average of 21.6 days. In the group with the last flushing performed >90 days prior, only two catheters had a positive culture. A catheter with a flushing interval of 110 days presented a positive culture for Enterococcus faecalis, and another catheter with a flushing interval of 601 days without flushing the device presented a positive culture for *Staphylococcus epidermidis.*

This finding reflects the possibility of colonization of fully implantable long-term catheters in patients without signs of infection, regardless of the maintenance of the flushing regimen that the service adopts for ports. Therefore, despite the surveillance period during which the patient retains the port even after the end of the oncological treatment, potential damage to the patient may still occur.

From a general point of view, of the five patients who had a positive catheter tip culture, three were in the group with <90 days, and two patients had flushing for >90 days, showing that the prolonged period of flushing and maintaining the catheters did not increase the risk of catheter contamination, nor did it protect it, as the reservoir was exposed to a lower number of punctures, as suggested. This finding was also noted by Ignatov, who observed no increase in infection at intervals greater than 8 weeks.^(12)^

In the case of a better quality of life made possible by a lower frequency of flushing, we must remember the socioeconomic profile of patients treated at *Hospital das Clínicas*. The cost of displacement is highlighted, with an average of R$ 39,00 (7,53 USD) just for one way and, therefore, a total of R$78,00 to go back and forth from the hospital on average. Considering the Brazilian minimum wage, this would be 6.4% of the salary required for this displacement.

Without considering the time needed to go to the hospital, with an average of 101 min or 1 h and 41 min, which greatly compromises the patient's quality of life, in addition to removing him from the working day.

Considering that the patients analyzed were treating breast cancer, whose period with a catheter for chemotherapy and then its surveillance totaled 5 years, or Hodgkin's lymphoma, whose patient remained with the catheter for 2 years, we can infer both the total monetary expenditure and the time required for displacement in this period. Thus, it is estimated that in the case of malignant breast cancer, 60 catheter washes were performed in the period (5 years × 12 months), with an expenditure of RS4.680,00 (904,44 USD) and 6,060 minutes just to go to the hospital. Patients with Hodgkin's lymphoma underwent a total of 24 washes (2 years × 12 months), which generated an expense of R$1,872.00 (361,77 USD) and 2,424 minutes dedicated to traveling to the hospital. With the stoppage of port maintenance flushing, which the COVID-19 pandemic has imposed on the service, both the cost and time devoted to such activities have decreased.

Additionally, the financial cost of commuting decreased and the quality of life for patients improved, with fewer visits for catheter flushing.

More studies are needed to evaluate the ideal period for maintenance flushing of ports, reconciling a lower incidence of complications, better cost-effectiveness, and a better quality of life for patients.

## CONCLUSION

Among patients >18 years of age being followed up at the Cancer Institute of the State of São Paulo during the COVID-19 pandemic, no statistically significant difference was found in the incidence of catheter colonization rate and malfunctioning of long-term catheters between those who underwent flushing with intervals of <90 days and those with intervals of >90 days. Patients who flushed for periods longer than 90 days experienced savings in time and money.
